# Structural Diversity of the Microbial Surfactin Derivatives from Selective Esterification Approach

**DOI:** 10.3390/ijms16011855

**Published:** 2015-01-15

**Authors:** Chuanshi Shao, Lin Liu, Hongze Gang, Shizhong Yang, Bozhong Mu

**Affiliations:** State Key Laboratory of Bioreactor Engineering and Institute of Applied Chemistry, East China University of Science and Technology, Shanghai 200237, China; E-Mails: 13641914007@163.com (C.S.); liulinkk@163.com (L.L.); ganghz@ecust.edu.cn (H.G.); shzhyang@ecust.edu.cn (S.Y.)

**Keywords:** Keywords: surfactin, esterification, derivatives, lipopeptide, carboxyl group

## Abstract

Surfactin originated from genus *Bacillus* is composed of a heptapeptide moiety bonded to the carboxyl and hydroxyl groups of a β-hydroxy fatty acid and it can be chemically modified to prepare the derivatives with different structures, owing to the existence of two free carboxyl groups in its peptide loop. This article presents the chemical modification of surfactin esterified with three different alcohols, and nine novel surfactin derivatives have been separated from products by the high performance liquid chromatography (HPLC). The novel derivatives, identified with Fourier transform infrared spectroscopy (FT-IR) and electrospray ionization mass spectrometry (ESI-MS), are the mono-hexyl-surfactin C14 ester, mono-hexyl-surfactin C15 ester, mono-2-methoxy-ethyl-surfactin C14 ester, *di*-hexyl-surfactin C14 ester, *di*-hexyl-surfactin ester C15, *di*-2-methoxy-ethyl-surfactin ester C14, *di*-2-methoxy-ethyl-surfactin ester C15, *di*-6-hydoxyl-hexyl-surfactin C14 ester and, *di*-6-hydoxyl-hexyl-surfactin C15 ester. The reaction conditions for esterification were optimized and the dependence of yields on different alcohols and catalysts were discussed. This study shows that esterification is one of the most efficient ways of chemical modification for surfactin and it can be used to prepare more derivatives to meet the needs of study in biological and interfacial activities.

## 1. Introduction

**Figure 1:**
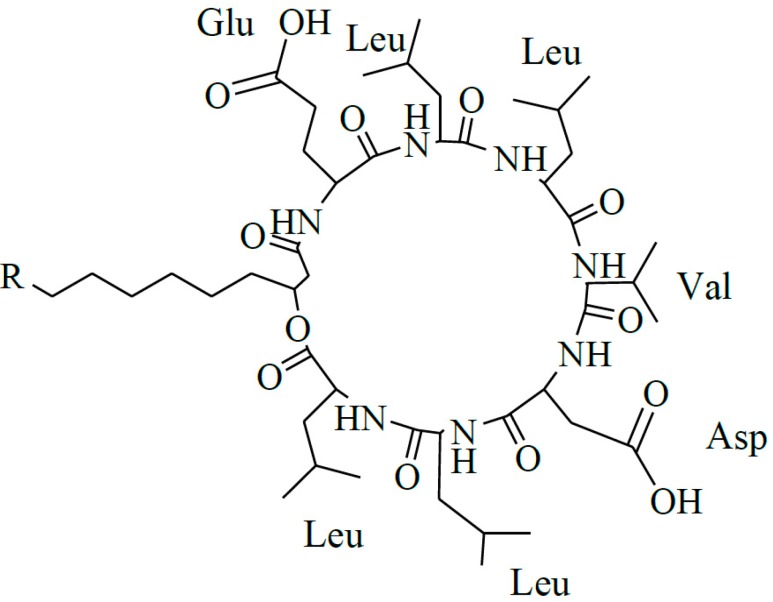
The structure of surfactin C13 (R = C_4_H_9_), C14 (R = C_5_H_11_), C15 (R = C_6_H_13_) [7].

Surfactin produced by strains of *Bacillus subtilis* is one of the most popular lipopeptide and has been studied for tens of years. It is a potent clotting inhibitor and can reduce the surface tension of water to 27 from 72 mN/m [1]. Surfactin is one of the cyclic lipopeptides lactonized by a heptapeptide and a β-hydroxy fatty acid. The typical heptapeptide is l-Glu-l-Leu-d-Leu-l-Val-l-Asp-d-Leu-l-Leu [2,3,4], although there exist some homologues differentiated in their peptide sequence, such as [Val7] surfactin, [Ile7] surfactin [5], [Ala4] surfactin [6], [Asp1, Glu5] surfactin [7]. The β-hydroxy fatty acids are *n*-, *iso*-, or *anteiso*-3-hydroxy fatty with 12–16 carbons [7,8,9]. The representative structure of surfactin is shown in Figure 1.

Much interest has been focused on the chemical modification of surfactin. At present, most reported modifications were concentrated on opening the lactone ring through hydrolysis, and on methylation of the peptide loop side chain. It was reported that the lactone ring could be opened by treatment with alkaline [10,11], the resulted linear surfactin lost 97% of the protoplast-bursting activity [10], had no significant hemolysis and the surface activity of it was reduced compared to the cyclic ones [12]. Considering about methylation, there are two possible approaches to get the methyl surfactin, chemical modification [2,4,13,14,15,16,17] and biosynthesis [18,19,20]. Because of the two carboxylic groups on side chains of the lactone ring, there would be different esterified derivatives. Surfactin-Glu-γ-methyl ester had a higher surfactant power than that of the surfactin, and a much higher haemolytic activity, 12 μM instead of 200 μM for 100% haemolysis [14], but lower anti-tumoral effect [18]. When residues of both an aspartic acid and a glutamic acid in surfactin were methylated (dimethyl-surfactin), the oil displacement activity increased by 20% and the derivatives showed an increased acid tolerance [16], but showed no virus-inactivation capacity [21]. The amidated surfactin had very similar properties of dimethyl-surfactin [16]. There were also some articles about total chemical synthesis, either in the liquid phase [22] or solid phase system [12,23,24,25] to get cyclic or linear surfactin and their analogues with low yields.

It could be inferred from the previous study that the increase of the hydrophobic of the polar domain could improve the surface activity of surfactin, but hydrolysis of the lactone ring would decrease the surface activity. Esterification is one of the most efficient ways of chemical modification [26]. In this article, we studied on producing variety of esterified surfactin derivatives in different reaction systems. The new compounds would provide some novel ideas to carry out study on the structure and property relationship of surfactin.

## 2. Results and Discussion

### 2.1. Identification of Surfactin-(Glu-γ, Asp-β)-Hexyl Ester

**Figure 2:**
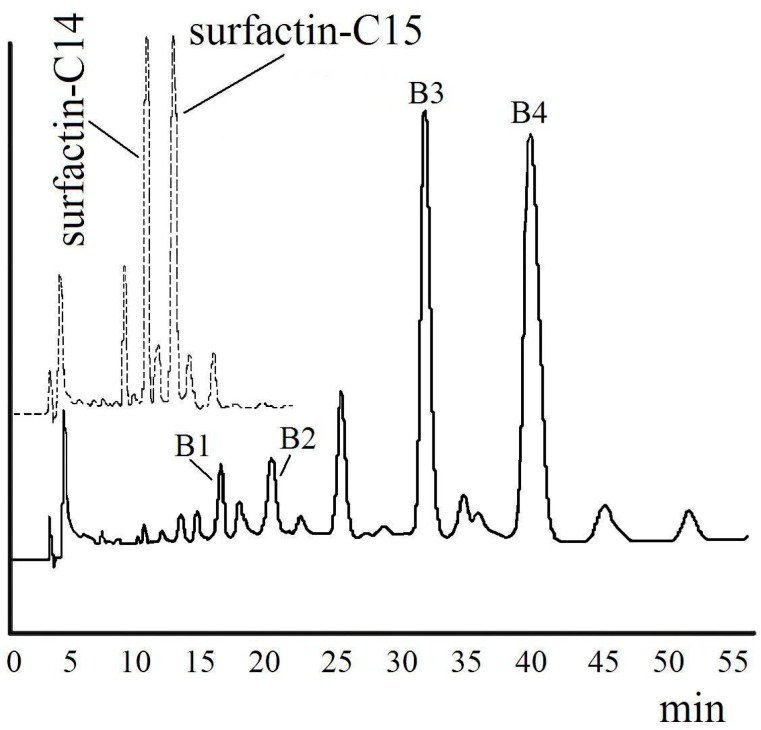
High performance liquid chromatography (HPLC) spectra of surfactin (dashed line) and surfactin-(Glu-γ, Asp-β)-hexyl ester (solid line) under the same condition.

The product formed by the reaction of surfactin with *n*-hexyl alcohol was purified by HPLC (High Performance Liquid Chromatography) and identified by FT-IR (Fourier Transform InfraRed Spectroscopy) and ESI-MS (Electrospray Ionization Mass Spectrometry). Four fractions B1, B2, B3 and B4 were separated, as shown in Figure 2. The yields of B1, B2, B3 and B4 were 3.4%, 5.4%, 32.4% and 48.2%, respectively.

**Figure 3:**
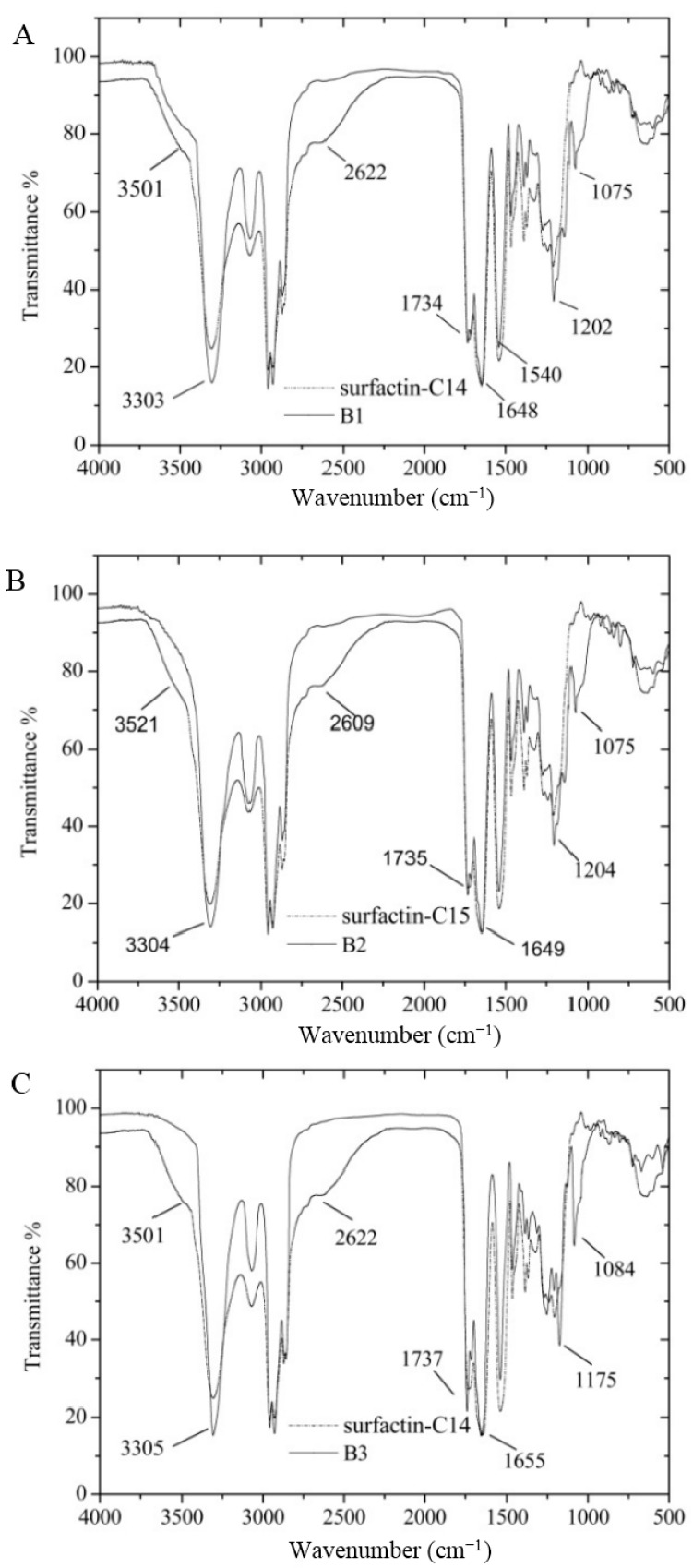

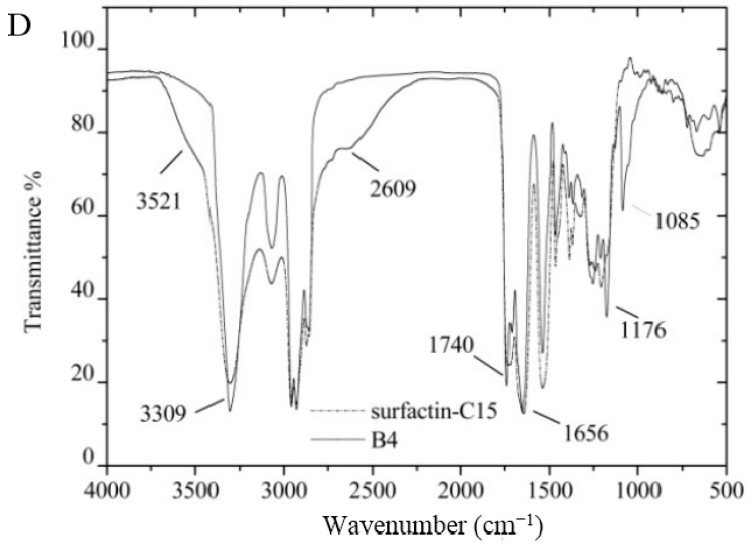
Fourier transform infrared spectroscopy (FT-IR) results of surfactin-C14, surfactin-C15, B1, B2, B3 and B4. (**A**) for surfactin-C14 and mono-hexyl-surfactin C14 ester; (**B**) for surfactin-C15 and mono-hexyl-surfactin C15 ester; (**C**) for surfactin-C14 and *di*-hexyl-surfactin C14 ester; and (**D**) for surfactin-C15 and *di*-hexyl-surfactin C15 ester.

FT-IR spectroscopy of B1–B4 was described in Figure 3. The FT-IR spectroscopy for purified compounds showed strong absorbance from 3500 to 3200 cm^−1^ with the maximum at 3303, 3304, 3305 and 3309 cm^−1^ for B1, B2, B3 and B4, respectively, which implies a typical feature stretching of N–H in the peptide. The maximum absorbance around 1650 cm^−1^ belonged to C=O stretching vibration of the amide I region. The interaction of C–N stretching mode of C–N–H group and N–H bending vibration contributed to the absorbance around 1550 cm^−1^. The absorbance between 3000 and 2800 cm^−1^ was the evidence of aliphatic chain. The absorbance among 1800–1700 cm^−1^ was due to the C=O stretching mode of the lactone ring. Therefore, all these four fractions, B1, B2, B3 and B4 had the structure character of lipopeptide. C–O–C stretching mode located around 1190 and 1080 cm^−^^1^ region. Compared with that of the original surfactin-C14 and surfactin-C15, the absorbance intensity of B1/B2 and B3/B4 rose significantly in these two regions, which indicated the new ester bonds were introduced after this modification process. O–H stretching had a broad absorbance from 3700–2200 cm^−1^, and the new compounds had narrow absorbance in this area revealed the reduction of carboxylic acid group. The FT-IR spectroscopy indicated that B1, B2, B3 and B4 were esterified surfactin.

**Figure 4:**
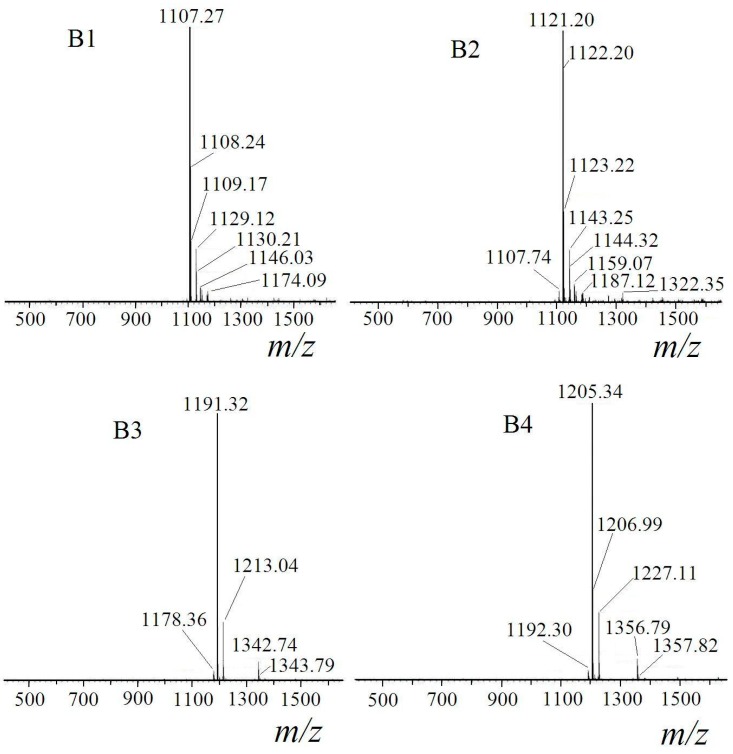
Electrospray ionization mass spectrometry (ESI-MS) spectroscopy of B1–B4.

The ESI-MS spectra of B1, B2, B3 and B4 showed the ionized molecular *m*/*z* in Figure 4. For B1, the major *m*/*z* peak was 1107, while *m*/*z* peak at 1129 could also be found in the same mass spectrum. The difference between the two values was 22. Considering the common positive ionization mode: [M + H]^+^, [M + Na]^+^ and [M + K]^+^, the modes for B1 were [M + H]^+^ and [M + Na]^+^. The molecular weight of B1 was 1106, which was equaled to molecular weight calculation value of [M_surfactin-C14_ + M_*n*-hexylalcohol_ − ${\text{M}}_{{\text{H}}_{2}\text{O}}$]. Combined with FT-IR results, B1 was mono-hexyl-surfactin-C14, which derived from surfactin-C14 with one of the carboxylic acid groups esterified by *n*-hexyl alcohol. The molecular weight of B2 was determined by the same way. B3 had the molecular weight of 1190, which equaled to the molecular weight calculation value of [M_surfactin-C14_ + 2 × M_*n*-hexylalcohol_ − 2 × ${\text{M}}_{{\text{H}}_{2}\text{O}}$]. The molecular weight of B4 was equaled to molecular weight calculation value of [M_surfactin-C15_ + 2 × M_*n*-hexylalcohol_ − 2 × ${\text{M}}_{{\text{H}}_{2}\text{O}}$].

**Figure 5:**
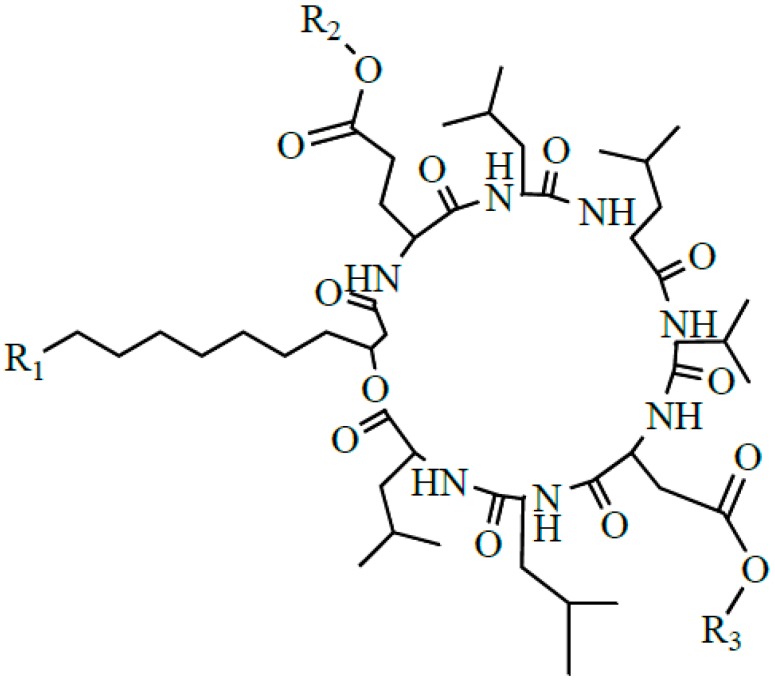
The structures of B1, B2, B3 and B4. B1: R_1_ = C_5_H_11_, R_2_ = C_4_H_9_, R_3_ = H or R_1_ = C_5_H_11_, R_2_ = H, R_3_ = C_6_H_13_; B2: R_1_ = C_6_H_13_, R_2_ = C_6_H_13_, R_3_ = H or R_1_ = C_6_H_13_, R_2_ = H, R_3_ = C_6_H_13_; and B3: R_1_ = C_5_H_11_, R_2_ = C_6_H_13_, R_3_ = C_6_H_13_; B4: R_1_ = C_6_H_13_, R_2_ = C_6_H_13_, R_3_ = C_6_H_13_.

The component identification results were listed in Table 1. B1 and B2 were mono-hexyl-surfactin ester, while B3 and B4 were *di*-hexyl-surfactin ester. The structures of B1, B2, B3 and B4 were described in Figure 5.

**Table 1:** Component identification results of B1, B2, B3 and B4.

Fraction	*m*/*z*	Ionization Mode	Molecular Weight	Compound
B1	1107	[M + H]^+ ^	1106	mono-hexyl-surfactin C14 ester
	1129	[M + Na]^+^		
B2	1121	[M + H]^+^	1120	mono-hexyl-surfactin C15 ester
	1143	[M + Na]^+^		
B3	1191	[M + H]^+^	1190	*di*-hexyl-surfactin C14 ester
	1213	[M + Na]^+^		
B4	1205	[M + H]^+^	1204	*di*-hexyl-surfactin C15 ester
	1227	[M + Na]^+^		

### 2.2. Identification of (Glu-γ, Asp-β)-2-Methoxy-Ethyl-Surfactin Ester

**Figure 6:**
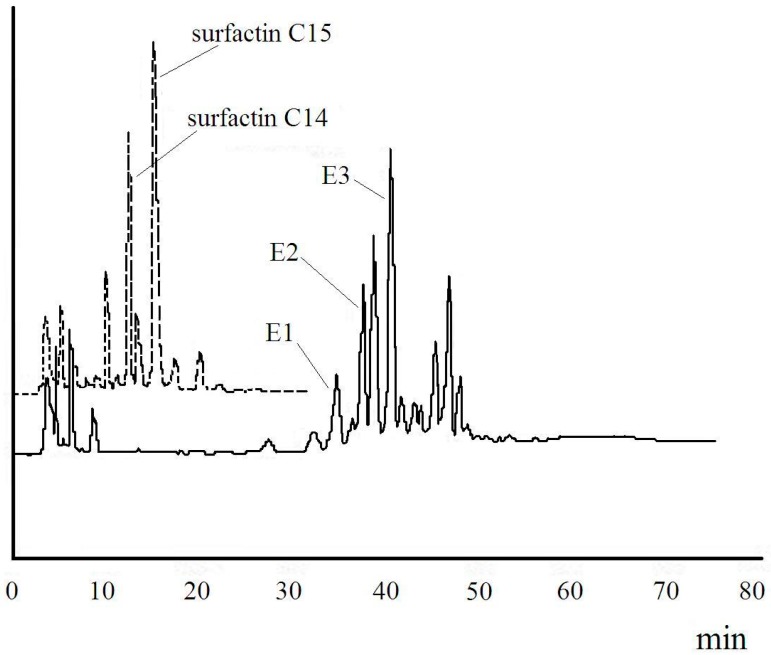
HPLC spectra of surfactin (dashed line) and (Glu-γ, Asp-β)-2-methoxy-ethyl-surfactin ester (solid line) under the same condition.

Analytical and preparative HPLC were used to analyze and separate the product from the esterification and surfactin with 2-methoxyethanol. Three fractions, E1, E2 and E3, were purified from the product, as shown in Figure 6. The yields of E1, E2 and E3 were 1.3%, 11.0% and 16.9%, respectively.

**Figure 7:**
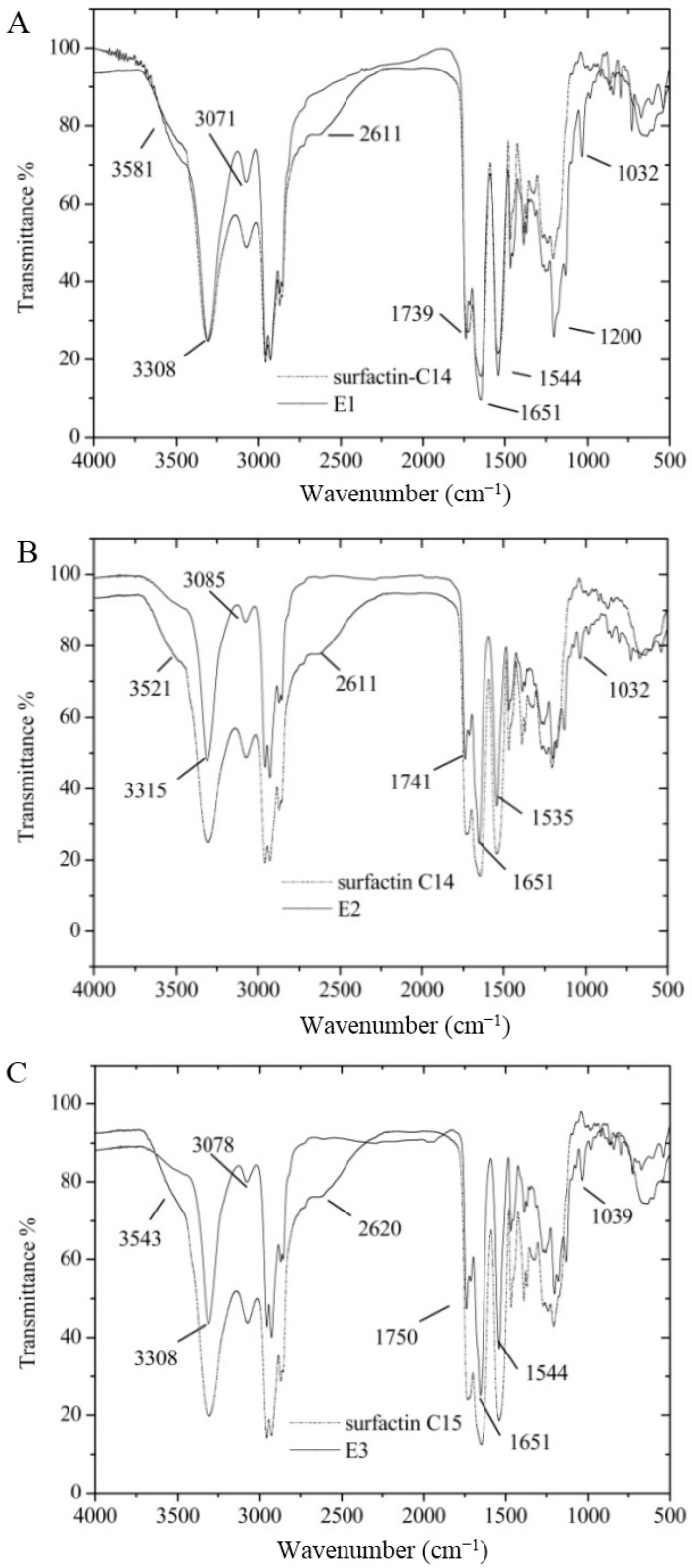
FT-IR spectra of E1, E2 and E3. (**A**) for the surfactin-C14 and mono-2-methoxy-ethyl-surfactin-C14 ester (E1); (**B**) for the surfactin-C14 and *di*-2-methoxy-ethyl-surfactin C14 ester; and (**C**) for surfactin-C15 and *di*-2-methoxy-ethyl-surfactin C14 ester.

FT-IR contradiction between original surfactin and reaction products was described in Figure 7. E1, E2 and E3 showed a significant characteristic transmittance of lipopeptide at the certain wave number. For E1, E2 and E3, there was no strong absorbance around 2610 cm^−1^, which might relate to carboxylic acid dimmer of Glu and Asp. The absorbance in 1032 cm^−1^ implied the new ester bond was introduced, which implied that E1, E2 and E3 were surfactin esters.

**Figure 8:**
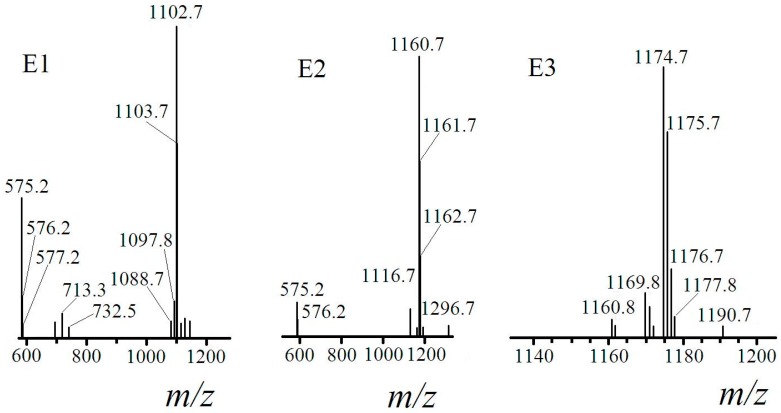
ESI-MS spectroscopy of E1, E2 and E3.

**Figure 9:**
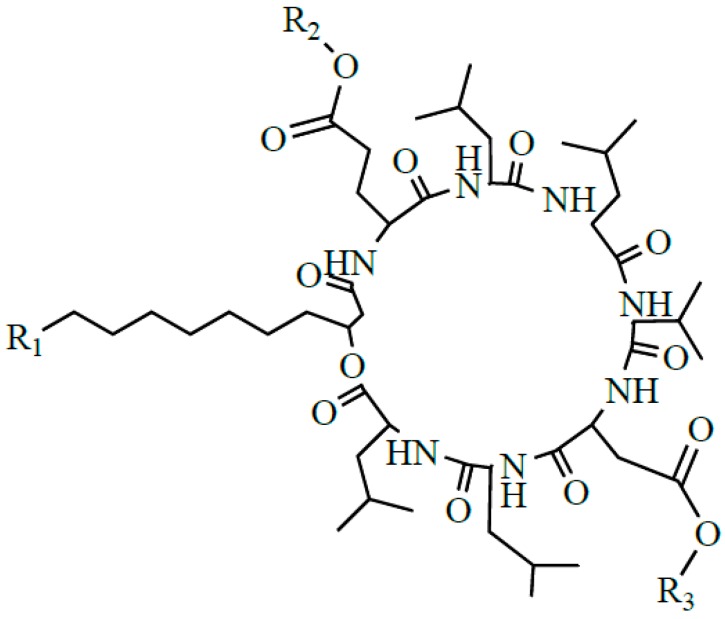
The structures of E1, E2 and E3. E1: R_1_ = C_5_H_11_, R_2_ = CH_2_–CH_2_–O–CH_3_, R_3_ = H or R_1_ = C_5_H_11_, R2 =H, R_3_ = CH_2_–CH_2_–O–CH_3_; E2: R_1_ = C_5_H_11_, R_2_ = CH_2_–CH_2_–O–CH_3_, R_3_ = CH_2_–CH_2_–O–CH_3_; and E3: R_1_ = C_6_H_13_, R_2_ = CH_2_–CH_2_–O–CH_3_, R_3_ = CH_2_–CH_2_–O–CH_3_.

E1, E2 and E3 had quasi-molecular ion peak, *m*/*z* at 1102.7, 1160.7 and 1174.7, respectively (Figure 8). The usual ionized mode in ESI-MS were [M + H]^+^, [M + Na]^+^ and [M + K]^+^. When the mode was assumed as [M + Na]^+^, the molecular weight of E1 was 1079.7, which equaled to the molecular weight calculation value of [M_surfactin-C14_ + M_2-methoxyethanol_ − ${\text{M}}_{{\text{H}}_{2}\text{O}}$]. E1 was the surfactin C14 ester with one of the carboxylic acid groups esterified by 2-methoxyethanol, which could be named as mono-2-methoxy-ethyl-surfactin C14 ester. The same analysis method was used to obtain the molecular weight of E2 and E3 (Table 2). Their structures were elaborated in Figure 9.

**Table 2:** Component identification results of E1, E2 and E3.

Fraction	*m*/*z*	Ionization Mode	Molecular Weight	Compound
E1	1102	[M + Na]^+^	1079	mono-2-methoxy-ethyl-surfactin C14 ester
E2	1160	[M + Na]^+^	1137	*di*-2-methoxy-ethyl-surfactin C14 ester
E3	1174	[M + Na]^+^	1151	*di*-2-methoxy-ethyl-surfactin C15 ester

### 2.3. Identification of Surfactin-(Glu-γ, Asp-β)-6-Hydoxyl-Hexyl Ester

**Figure 10:**
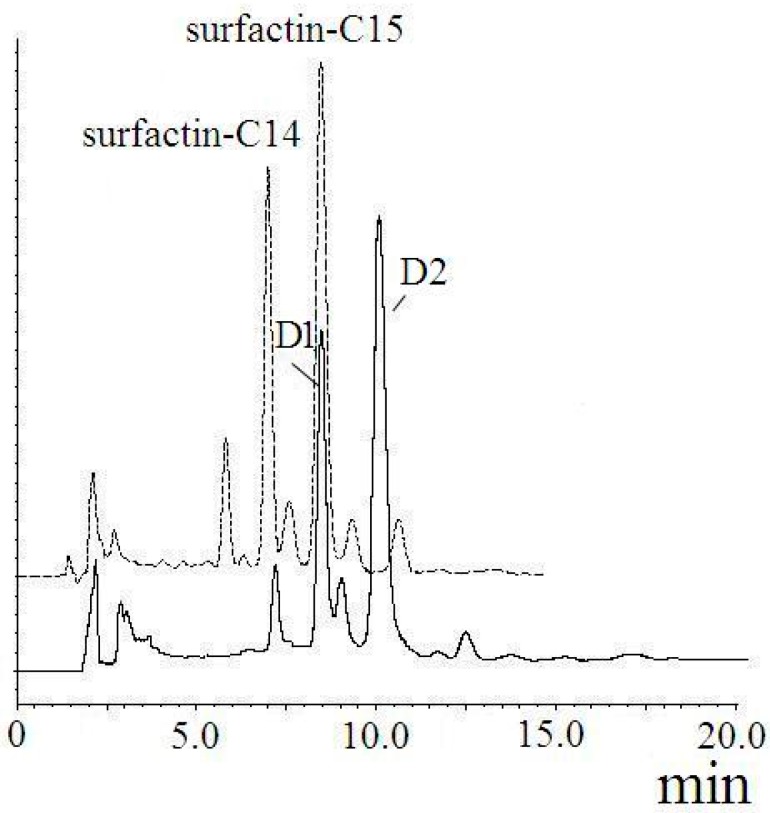
HPLC spectra of surfactin (dashed line) and (Glu-γ, Asp-β)-6-hydoxyl-hexyl-surfactin ester (solid line).

The product obtained from reaction between surfactin and 1,6-hexanediol contained two major components, D1 and D2 (Figure 10). They were purified by semi-preparative HPLC under the above-mentioned condition. The yields of D1 and D2 were 33.8% and 53.1%, respectively.

**Figure 11:**
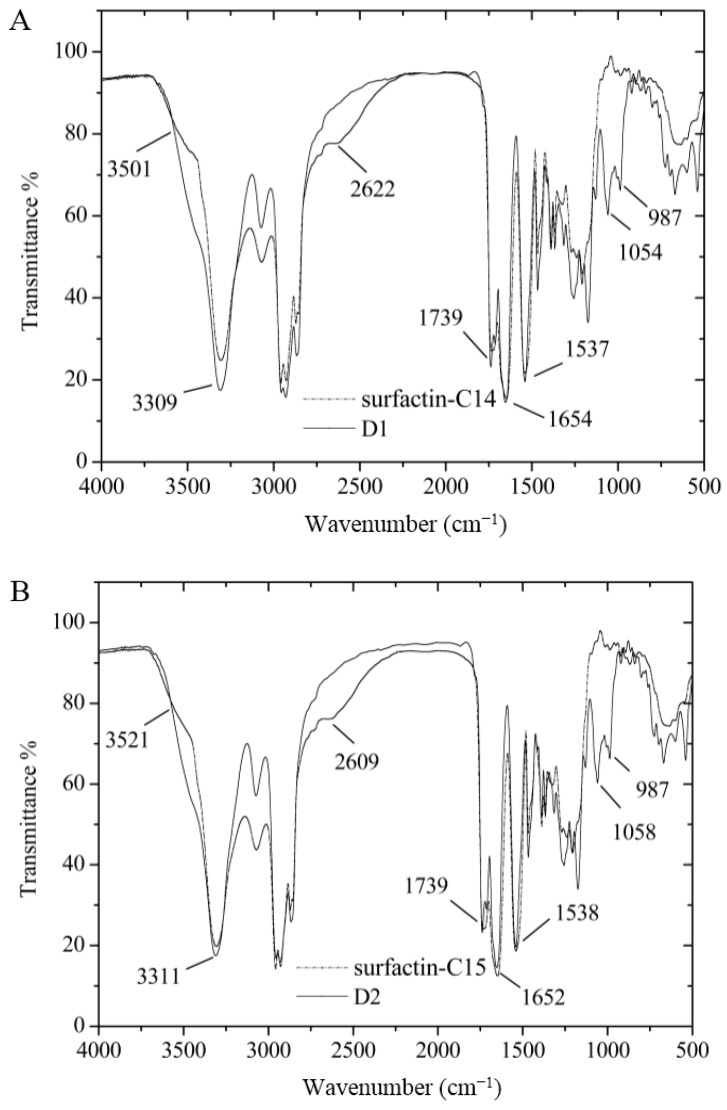
FT-IR results of surfactin-C14 and surfactin-C15, D1 and D2. (**A**) for surfactin-C14 and *di*-6-hydoxyl-hexyl-surfactin C14 ester; and (**B**) for surfactin-C15 and *di*-6-hydoxyl-hexyl-surfactin C15 ester.

FT-IR spectroscopy of D1 and D2 were described in Figure 11. The FT-IR spectroscopy for purified compounds showed the maximum absorbance at 1654 and 1652 cm^−1^ belonging to C=O stretching vibration of the amide I region. And there was strong absorbance from 3500 to 3200 cm^−1^ with the maximum at 3309 and 3311 cm^−1^ for D1 and D2, respectively, which was a typical feature stretching of N–H in the peptide. The absorbance at 1537 and 1538 cm^−1^ was due to the interaction of C–N stretching mode of C–N–H group and N–H bending vibration. The absorbance between 3000 and 2800 cm^−1^ was the evidence of aliphatic chain. The absorbance around 1730 cm^−1^ was due to the C=O stretching mode of the lactone ring. Therefore, it could be confirmed that D1 and D2 had the structure character of lipopeptide. Compared with that of the original surfactin-C14 and surfactin-C15, the absorbance intensity rose significantly at 1054 cm^−^^1^ for D1 and at 1058 cm^−^^1^ for D2. Those two regions related to C–O–C stretching mode, which proved new ester bonds were introduced. From 3700 to 2200 cm^−^^1^, the broad absorbance of O–H stretching still remained for D1 and D2, but more narrow compared with that of original surfactin. The new absorbance of 987 cm^−^^1^ represents the interaction between (O–H) bending vibration and (C=O) stretching. So hydroxyl group existed in both D1 and D2. It could be indicated that D1 and D2 were esterified surfactin with hydroxyl group.

**Figure 12:**
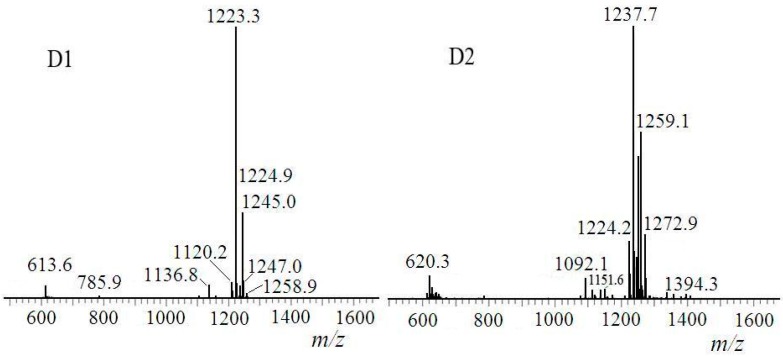
ESI-MS spectroscopy of D1 and D2.

**Figure 13:**
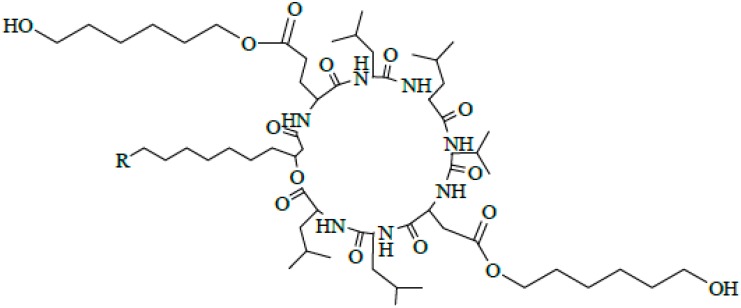
The structures of D1 and D2. D1: R = C_5_H_11_; D2: R = C_6_H_13_.

ESI-MS of D1 and D2 was shown in Figure 12. The molecular weight of D1 was revealed to be 1222, for [M + H]^+^ ion at *m*/*z* 1223 and [M + Na]^+^ ion at *m*/*z* 1245 accordingly, which equaled to molecular weight calculation value of [M_surfactin-C14_ + 2 × M_1,6-hexanediol_ – 2 × ${\text{M}}_{{\text{H}}_{2}\text{O}}$]. D1 was *di*-6-hydoxyl-hexyl-surfactin ester C14. Component identification results were listed in Table 3. Both D1 and D2 were *di*-6-hydoxyl-hexyl-surfactin ester, which structures were described in Figure 13.

**Table 3:** Component identification results of D1 and D2.

Fraction	*m/z*	Ionization Mode	Molecular Weight	Compound
D1	1223	[M + H]^+^	1222	*di*-6-hydoxyl-hexyl-surfactin C14 ester
	1245	[M + Na]^+^		
D2	1237	[M + H]^+^	1236	*di*-6-hydoxyl-hexyl-surfactin C15 ester
	1259	[M + Na]^+^		

### 2.4. Discussion

The typical surfactins are the two homological compounds, C14 and C15, and the difference between them is the length of the hydrophobic chain. New compounds B1, B3 and D1 were derived from surfactin-C14, while B2, B4 and D2 from surfactin-C15. HPLC chromatogram pattern had highly similarity between the original surfactins and the esterified ones. In the original surfactin, component ratio of C14 and C15 was approximately 1:1.57, while the yield one of (B1 + B3): (B2 + B4) equaled to 1: 1.50, E2:E3 equaled to 1:1.54 and D1:D2 equaled to 1:1.57. It implies that the individual derivative yields of C14 and C15 were directly related to the ratio of each component in the original surfactin.

Since there are two carboxyl groups in the peptide moiety of surfactin, their chemically modified products exhibit two classes of forms. One is either Asp-β- or Glu-γ-carboxyl group esterified by alcohol (monoester-SF), B1, B3 and E1, while the other is both of the two carboxyl groups esterified (diester-SF), B2, B4, E2, E3, D1 and D2. Owing to the composition of the surfactin and esterification mode, modified compounds were diverse in form. The structural diversity of the surfactin derivatives indicates their difference in properties.

We had tried to use concentrated HCl to catalyze the reaction of surfactin with 2-methyethanol and 1,6-hexanediol, but the results were not satisfied as there was almost 60% original surfactin still remained in the reaction solvent after stirred for over 4 days. It is probably because of the existence of two hydroxyl groups or polyoxyethylene group in the same molecule reduced the individual hydroxyl group electron density, and therefore decreased the reactivity of 2-methyethanol and 1,6-hexanediol. DCC/DMAP [27] and EDC/DMAP [28] were common catalysts for esterification, but based on current references, these two catalysts had not been applied to surfactin esterification. It was found that the product from DCC/DMAP catalysis contained some byproducts, which is because of the 1,3-rearrangement of intermediate *O*-acylisoura leading to the forming of *N*-acylura that could not react with alcohol. The yield of byproducts was above 30%. While after reacted in the EDC/DMAP system for 24 h, surfactin was completely transferred into ester. Based on current work, the merit of EDC/DMAP catalyzing esterification method was fast and complete.

**Figure 14:**
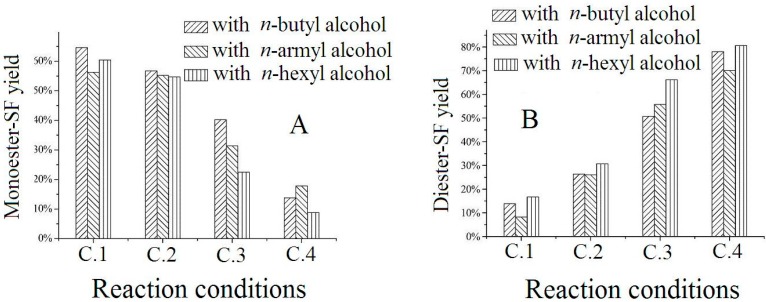
The ratio of (**A**) monoester-SF and (**B**) diester-SF in the product in four reaction conditions. Condition 1: catalyzed by 1 mol/L HCl; reacted for 24 h; Condition 2: catalyzed by 1 mol/L HCl; reacted for 48 h; Condition 3: catalyzed by concentrated HCl; reacted for 24 h; Condition 4: catalyzed by concentrated HCl; reacted for 48 h.

For HCl catalysis system, it was found that the ratios of monoester-SF and diester-SF in the products varied with the concentration of HCl and the reaction time. Three kinds of *n*-alcohol were selected to study the relationship between reaction condition and moiety type. Four conditions had been adopted to find regular pattern of the yield, condition 1 (C.1: catalyzed by 1 mol/L HCl; reacted for 24 h), condition 2 (C.2: catalyzed by 1 mol/L HCl; reacted for 48 h), condition 3 (C.3: catalyzed by concentrated HCl; reacted for 24 h) and condition 4 (C.4: catalyzed by concentrated HCl; reacted for 48 h). The ratios of monoester-SF and diester-SF in these four reaction conditions were plotted in Figure 14. The ratio for monoester-SF in the products was C.1 > C.2 > C.3 > C.4, while the ratio order was on the contrary for diester-SF. When the reaction was carried out in the 1 mol/L HCl, it was more inclined to create monoester-SF. For diester-SF concentrated HCl was preferred. Prolonging reaction time from 24 to 48 h was advantageous for the production of diester-SF, but disadvantageous for monoester-SF, which was consistent with Thimon’s report [14]. The results indicated that it was possible to regulate the ratio of surfactin monoester and diester, as well as to obtain specific derivative.

Compared with the original surfactin, the retention time of those new compounds on the reverse phase column was elongated, which suggested the improvement of the hydrophobic property of them. Furthermore, in our experiment condition, B4 and D2 had nano pore phenomenon with the artificial bilayer membrane with only one side addition of them to the membrane, while original surfactin should be added on both sides to show such phenomenon [29]. This related to the importance of hydrophobic interaction in molecular penetration capability [11].

The calcium tolerance ability and the oil displacement activity [16] were improved through the modifications since Glu and Asp are negative charged amino acid residues in alkaline solutions and easily chelated with calcium [30].

## 3. Experimental Section

### 3.1. Reagents

The surfactin sample we used was obtained from the cell-free broth of *Bacillus subtilis* HSO 121 in our lab. The *n*-hexyl-alcohol, *n*-butyl-alcohol, *n*-amyl-alcohol, 2-methoxyethanol, dicyclohexylcarbodiimide (DCC), 1-(3-dimethylaminopropyl)-3-ethylcarbodiimide hydrochloride (EDC), 4-dimethylaminopyridine (DMAP) were purchased from Aladdin-reagent Incorporation (Shanghai, China). The 1,6-hexanediol was purchased from Sigma-Aldrich (St. Louis, MO, USA).

### 3.2. Preparation of (Glu-γ, Asp-β)-Hexyl-Surfactin Ester

Fifty milligrams of surfactin was dissolved in 75 mL *n*-hexyl alcohol. Being added with 0.5 mL concentrated HCl, the solution was stirred at room temperature for 48 h and then successively washed by 0.5 mL redistilled water for three times to remove HCl, and dried with Na_2_SO_4_. After removing the residual alcohol under vacuum, the (Glu-γ, Asp-β)-hexyl-surfactin ester was prepared and applied to the analytical HPLC to monitor the components of the product. The purification was performed by RP-semi-preparative HPLC. The molecular weight and functional groups of product were detected by ESI-MS and FT-IR, respectively.

### 3.3. Preparation of (Glu-γ, Asp-β)-2-Methoxy-Ethyl-Surfactin Ester

One hundred milligrams of surfactin was dissolved in 100 mL chloroform and added with 50 mg DCC/5 mg DMAP and 3 mL 2-methoxyethanol in the ice-water bath. The reaction solution was stirred at room temperature for 24 h and then washed orderly by 100 mL fresh 1% citric acid solution, 100 mL 1% sodium bicarbonate solution, 100 mL redistilled water, 100 mL saturated salt water and 100 mL redistilled water, and retention organic phase was dried with Na_2_SO_4_. The organic solvent was filtered with 0.22 μm filter membrane before the chloroform removed under vacuum. After (Glu-γ, Asp-β)-2-methoxy-ethyl-surfactin ester was prepared, reverse phase HPLC was used to analyze the components of this product. Then the purification was achieved by RP-semi-preparative HPLC. The molecular weight and functional groups of product were separately detected by ESI-MS and FT-IR.

### 3.4. Preparation of (Glu-γ, Asp-β)-6-Hydoxyl-Hexyl-Surfactin Ester

One gram of surfactin was dissolved in 100 mL chloroform and added with 0.45 g EDC/0.29 g DMAP and 4.7 g 1,6-hexanediol in the ice-water bath, the reaction solution was stirred at room temperature for 24 h and then washed orderly by 100 mL fresh 1% citric acid solution, 100 mL 1% sodium bicarbonate solution, 100 mL redistilled water, 100 mL saturated salt solution and 100 mL redistilled water, and retained organic phase was dried with Na_2_SO_4_. After removing the chloroform under vacuum, (Glu-γ, Asp-β)-6-hydoxyl-hexyl-surfactin ester was prepared. Analytical HPLC was used to monitor the components of this product. And the purification was achieved by semi-preparative HPLC. ESI-MS and FT-IR could be used for the product molecular weight and functional group detection.

### 3.5. Analysis and Purification

Analytical HPLC was performed to determine the number of fractions on HPLC system (PU2080 Solvent Delivery System, CO-2060 Column Thermostat, 20 µL Quantitative Loop, UV-2075 Detector at 214 nm; Jasco Corporation, Tokyo, Japan). The chromatographic system equipped with a Hypersil ODS (Dalian Elite Analytical Instruments Co., Ltd., Dalian, China) C18 (Φ 4.6 mm × 25 cm) column which was eluted with isocratic gradient (A/B = 90/10; A: CH_3_OH, B: H_2_O + 0.05% TFA, 1 mL/min). Semi-preparative HPLC was also performed for purification on the same HPLC system under the same ratio of mobile phase while with an YMC ODS (YMC Co., Ltd., Kyoto, Japan) C18 (Φ 20 mm × 25 cm), 2 mL Quantitative Loop and the flow rate was 12 mL/min.

### 3.6. Molecular Weight Determination

ESI-MS was used for the molecular weight determination by LCQ Deca XP Plus ion trap mass spectrometer (Thermo Finnigan Co., San Jose, CA, USA), positive ion detection mode; ion source spray voltage 4.8 kV, capillary temperature 320 °C, capillary voltage 15 V, sheath gas nitrogen, flow rate 50 arb, auxiliary gas flow rate 20 arb, collision gas helium, test mode full scan mass rage of 50–2000 D.

### 3.7. Certification of Characteristic Functional Groups

The Fourier transform infrared spectroscopy (FT-IR) of purified surfactin derivatives were recorded by a Nicolet 6700 Fourier Transform Infrared Spectrophotometer (Thermo Fisher Scientific Inc., Waltham, MA, USA). The sample film was obtained by spreading 20 μL CH_2_Cl_2_ solution of 1 mg purified compound on the KBr crystal slice and drying under infrared light. The spectra were recorded by transmittance mode in a range of 4000–500 cm^−1^.

## 4. Conclusions

Three catalysis reaction systems were developed to study the esterification of surfactin and different surfactin derivatives have been obtained, which shows that the esterification is one of the most efficient ways of chemical modification for surfactin. The yields and type of the derivatives gave a dependence on the corresponding alcohol and catalyst. Three mono-esters (mono-hexyl-surfactin ester C14, mono-hexyl-surfactin ester C15, mono-2-methoxy-ethyl-surfactin ester C14), and six *di*-esters (*di*-hexyl-surfactin ester C14, *di*-hexyl-surfactin ester C15, *di*-2-methoxy-ethyl-surfactin ester C14, *di*-2-methoxy-ethyl-surfactin ester C15, *di*-6-hydoxyl-hexyl-surfactin ester C14, *di*-6-hydoxyl-hexyl-surfactin ester C15) have been obtained. HCl can catalyze the reaction of surfactin with *n*-alcohol to prepare both mono-ester and di-ester, and the ratio of those two products can be controlled by the adjustment of HCl concentration and reaction time. DCC/DMAP is an effective catalyst for surfactin esterification, but in this case the side reaction may affect the yield. It also shows that EDC/DMAP is the optimal catalyst for *di*-ester.
